# Rezūm Therapy in Very Elderly Men With BPH: Two‐Year Outcomes From a Multicenter Cohort

**DOI:** 10.1111/luts.70043

**Published:** 2025-12-28

**Authors:** Mario Henrique Bitar Siqueira, Sagi Shprits, Naeem Bhojani, Bilal Chughtai, Kevin C. Zorn, Luca Cindolo, Giovanni Ferrari, Katherine Lajkosz, Dean Elterman

**Affiliations:** ^1^ Division of Urology, Department of Surgery University of Toronto Toronto Canada; ^2^ Division of Urology Centre Hospitalier de l'Université de Montréal (CHUM) Montréal Canada; ^3^ Plainview Hospital Smith Institute of Urology, Northwell Health Syosset New York USA; ^4^ BPH Canada Prostate Surgical Institute Montreal Canada; ^5^ Urology Department Hesperia Hospital, CUrE Group Modena Italy; ^6^ Department of Biostatistics University Health Network Toronto Ontario Canada

**Keywords:** benign prostatic hyperplasia, elderly patients, functional outcomes, lower urinary tract symptoms, minimally invasive surgical therapy (MIST), water vapor thermal therapy

## Abstract

**Objectives:**

Rezūm water vapor thermal therapy is a minimally invasive surgical option with demonstrated efficacy and safety. However, real‐world evidence on 2‐year outcomes in the population aged above 80 years remains limited.

**Methods:**

A prospective registry was created as part of the International Rezūm Registry database at two high‐volume centers. Data were reviewed from patients monitored between April 2019 and August 2024. Men aged ≥ 80 years who underwent Rezūm therapy were included. Baseline and follow‐up assessments at 3, 6, 12, and 24 months included International Prostate Symptom Score (IPSS), IPSS Quality of Life (QoL) subscore, peak urinary flow rate (Qmax), and post‐void residual volume (PVR). Safety outcomes and adverse events were also recorded.

**Results:**

Fifty‐eight patients (mean age 84.4 ± 4.4 years) were included, with a mean prostate volume of 80.1 ± 42.1 cc. The mean IPSS decreased from 19.8 ± 7.4 at baseline to 12.4 ± 7.9 at 24 months. The IPSS quality of life (QoL) score declined from 4.2 ± 1.8 at baseline to 2.6 ± 1.5 at the corresponding follow‐up interval. Qmax improved from 8.5 ± 5.8 mL/s at baseline to 15.0 ± 4.0 mL/s at 24 months, respectively. PVR was reduced from 115.5 ± 93.7 mL at baseline to 42.8 ± 29.2 mL over the same period. However, neither Qmax nor PVR demonstrated a statistically significant change at any time point. Adverse events were infrequent, with urinary tract infections (8.6%), epididymitis (5.2%), and acute urinary retention (3.4%). Only one patient required hospitalization.

**Conclusion:**

Rezūm water vapor thermal therapy is a safe and effective treatment for BPH in patients over 80 years of age. It results in durable improvements in urinary symptoms and function with a low rate of complications.

## 1 Introduction

The global prevalence of benign prostatic hyperplasia (BPH) has risen markedly, increasing from an estimated 51.1 million cases in 2000 to 94.0 million in 2019 [1]. After the age of 50, 50% to 70% of men experience lower urinary tract symptoms (LUTS), with prevalence rising to 80%–90% in those over 80 years, primarily due to benign prostatic obstruction secondary to BPH [2]. In affected individuals, LUTS significantly impairs quality of life, underscoring the importance of timely diagnosis and effective management, particularly in the context of BPH [3].

While conservative management and pharmacologic therapy are typically the first‐line approaches for benign prostatic hyperplasia (BPH), some patients may experience adverse effects or persistent, refractory symptoms, ultimately requiring surgical intervention [4]. Over the past decades, a variety of treatment modalities for lower urinary tract symptoms (LUTS) secondary to benign prostatic obstruction (BPO) have been introduced, offering varying degrees of invasiveness and efficacy [5]. One of the most recent minimally invasive surgical therapies is radiofrequency‐generated water vapor thermal therapy, known as the Rezūm System (Boston Scientific, Marlborough, MA, USA).

Rezūm thermal therapy is distinguished by its ability to treat all prostate zones, regardless of anatomical morphology. This is particularly important, as intravesical prostatic protrusions are now recognized as predictors of poor outcomes with most pharmacologic treatments and are often associated with urodynamic obstruction [6]. Its ability to provide rapid and durable relief of LUTS, while improving quality of life and maintaining low rates of sexual dysfunction, makes it an outstanding treatment option [7]. While the versatility of Rezūm across various prostate anatomies is well established, there is still a lack of robust investigation regarding its outcomes specifically in older adults.

Geriatric patients, who are more susceptible to adverse events (AEs) and often exhibit poor adherence to pharmacologic treatments, may benefit from minimally invasive surgical therapies (MISTs) for the management of benign prostatic hyperplasia (BPH) [8]. Aging is also associated with a decrease in the bladder smooth muscle‐to‐collagen ratio, which can impair contractile function [9]. Furthermore, bladder innervation tends to decline over time, particularly in the presence of chronic bladder overactivity or outlet obstruction [10].

A study assessing the prevalence of lower urinary tract symptoms (LUTS) in patients aged ≥ 80 versus < 80 years revealed a significantly higher symptom burden in the older cohort, particularly regarding incomplete bladder emptying, increased frequency, urgency, and weak urinary stream [11]. This study aims to further explore urinary function and safety specifically in patients over 80 years of age who have undergone the Rezūm procedure.

## 2 Material and Methods

### 2.1 Study Subjects

This study is a retrospective review of a prospectively collected database from two high‐volume centers performing water vapor thermal therapy (WVTT–Rezūm) in Canada and Italy. Institutional ethics board approval was obtained at each center. The cohort included patients who underwent Rezūm therapy between April 2019 and August 2024, with eligibility restricted to those aged 80 years and older. Twelve patients (20.7%) were catheter‐dependent with a Foley catheter prior to Rezūm; no patients were on CIC. Procedures were performed under either local anesthesia with intravenous sedation or spinal anesthesia, based on patient preference and anesthesiologist assessment. Propofol‐based intravenous sedation was used in 42 cases (72.4%), while spinal anesthesia was administered in 16 cases (27.6%).

Rezūm water vapor thermal therapy was performed according to previously established methods [12]. Each application of water vapor ablated contiguous regions of prostatic tissue, following the natural course of the urethra. The number of injections was determined at the surgeon's discretion based on the patient's prostatic anatomy and the goal of preserving ejaculation. As the Rezūm system allows a maximum of 15 injections per disposable device, procedures requiring additional injections necessitate the use of a corresponding number of devices. Patients were discharged the same day with a Foley catheter in place. The duration of catheterization ranged from 7 to 30 days, depending on the presence of urinary retention.

Perioperative bleeding was categorized into three grades: Grade 1, minimal and self‐limiting bleeding that did not require intervention; Grade 2, moderate bleeding managed with conservative measures such as catheter irrigation or topical hemostatic agents, but not requiring transfusion or surgical intervention; and Grade 3, severe bleeding necessitating blood transfusion and/or surgical or endoscopic intervention.

### 2.2 Data Collection and Outcomes of Interest

Patients were evaluated at baseline, and at 3‐, 6‐, 12‐, and 24‐months post‐treatment. Collected data included patient demographics, prostate volume, number of Rezūm injections, procedure‐related complications, and BPH medication use at baseline and follow‐up. Clinical assessments included the International Prostate Symptom Score (IPSS) and its Quality of Life (QoL) subscale, maximum urinary flow rate (Qmax), and post‐void residual volume (PVR).

The primary outcome was the effectiveness of Rezūm therapy in improving urinary symptoms and objective measures of urinary function, as assessed by IPSS, IPSS QoL, Qmax, and PVR. These outcomes were evaluated using baseline and follow‐up data collected throughout the study period. Secondary outcomes focused on treatment safety and adverse events.

### 2.3 Statistical Analysis

Patient characteristics and outcomes were analyzed descriptively. Continuous variables were compared using Student's *t*‐test or rank‐sum tests, depending on data distribution. Categorical variables were analyzed using Fisher's exact test. All statistical analyses were conducted using Stata 18BE, with a two‐sided *p* value < 0.05 considered statistically significant.

## 3 Results

A total of 58 patients who underwent the Rezūm procedure were included in this analysis. The mean age at the time of the procedure was 84.4 years (SD: 4.4). The mean baseline prostate volume was 80.1 cc (SD: 42.1). A median lobe was present in 36 patients (62.1%). The mean duration of Foley catheterization was 13.5 ± 8.8 days (range, 3–30 days). Among patients aged ≤ 89 years (*n* = 51), the mean Foley duration was 12.8 ± 8.3 days with a median of 7 days, whereas in those aged ≥ 90 years (*n* = 7), the mean duration was 16.1 ± 11.9 days with a median of 10 days. Although patients aged ≥ 90 years tended to have a slightly longer mean and a wider range of Foley use, this difference was not statistically significant (*p* = 0.71). Pre‐procedure urinary management included the use of a Foley catheter for chronic urinary retention in 12 patients (20.7%). Regarding medication use, 18 patients (31%) were on alpha‐blockers, and 18 patients (31%) were on 5‐alpha reductase inhibitors. After the procedure, only two patients continued the medications they had been taking prior to treatment—one remained on an alpha‐blocker and the other on a PDE‐5 inhibitor. The procedure involved a mean of 10.5 vapor injections (SD: 5.0). The mean duration for scope in‐and‐out was 4.1 min (SD: 2.3) (Table 1).

**TABLE 1:** Baseline and procedural characteristics (*n* = 58).

Variable	Value
Age at Rezūm (years)	
Mean (SD)	84.4 (4.4)
Median (Q1–Q3)	83.1 (81.2–86.7)
Range (min–max)	80.0–100.7
Median lobe	36 (62.1%)
Hypertension	28 (51.9%)
Diabetes	3 (5.6%)
History of urinary retention	19 (32.8%)
Baseline prostate volume (mL)	
Mean (SD)	80.1 (42.1)
Median (Q1–Q3)	65.0 (53.0–99.8)
Range (min–max)	20.0–195.0
Current Foley catheter use	12 (20.7%)
Alpha blockers	18 (31.0%)
5‐ARI inhibitors	18 (31.0%)
Anticoagulants/Antiplatelets	19 (32.8%)
Total number of vapor injections	
Mean (SD)	10.5 (5.0)
Median (Q1–Q3)	10.0 (6.2–13.0)
Range (min–max)	3–28
Scope in–out duration (min)	
Mean (SD)	4.1 (2.3)
Median (Q1–Q3)	3.5 (3.0–5.0)
Range (min–max)	1–11
Bleeding scale	
Mean (SD)	1.1 (0.4)
Median (Q1–Q3)	1 (1–1)
Range (min–max)	1–3

*Note:* Baseline demographics, clinical characteristics, and perioperative procedural details of the study cohort.

Abbreviations: 5‐ARI, 5‐α reductase inhibitor; min–max, minimum–maximum; Q1–Q3, interquartile range; SD, standard deviation.

### 3.1 Urinary Status

**FIGURE 1:**
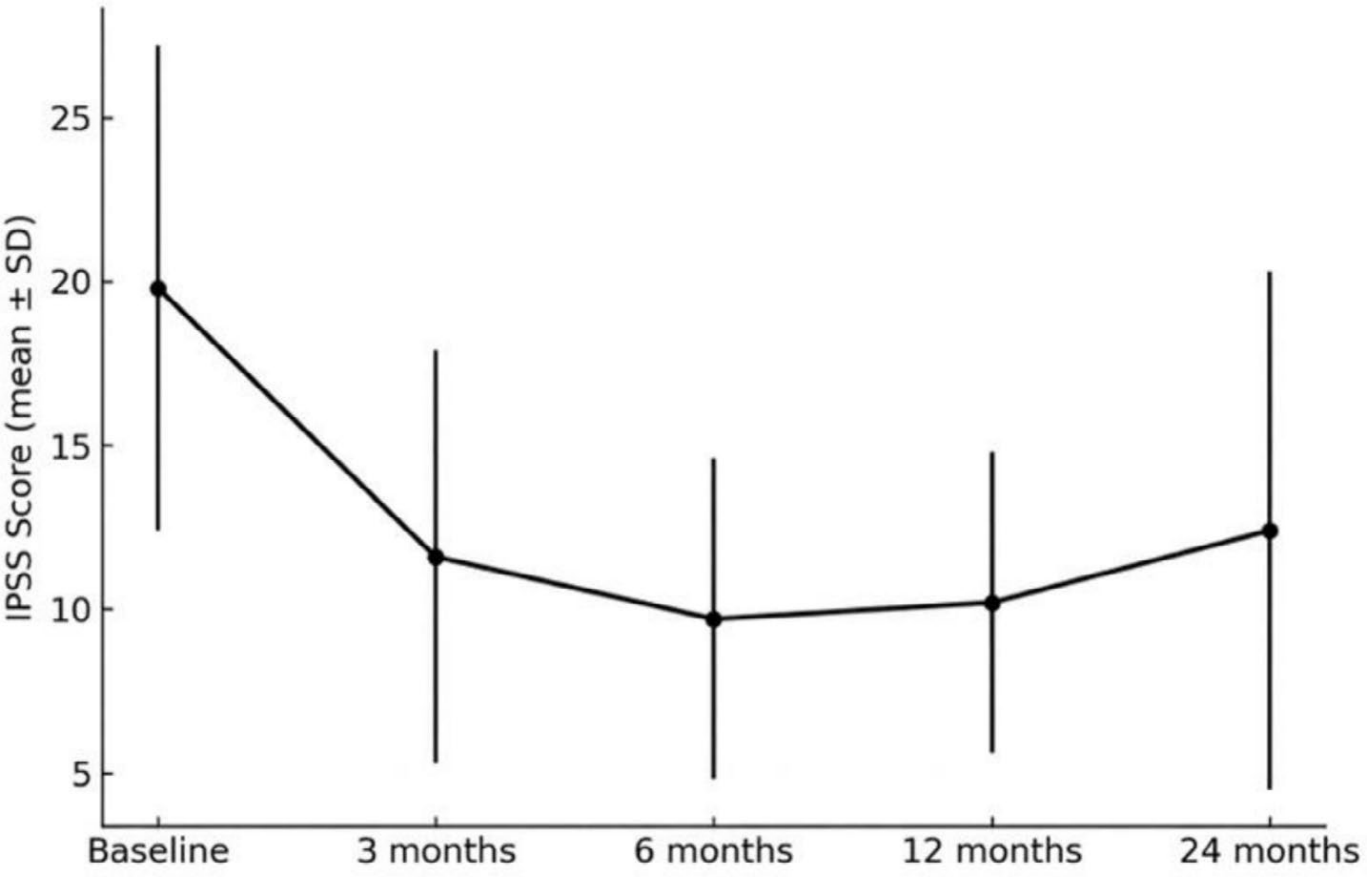
IPSS**—**International prostate symptom score. IPSS over time following Rezūm therapy (mean ± SD); *p* < 0.005 versus baseline at 3‐, 6‐, 12‐, and 24‐months.

**FIGURE 2:**
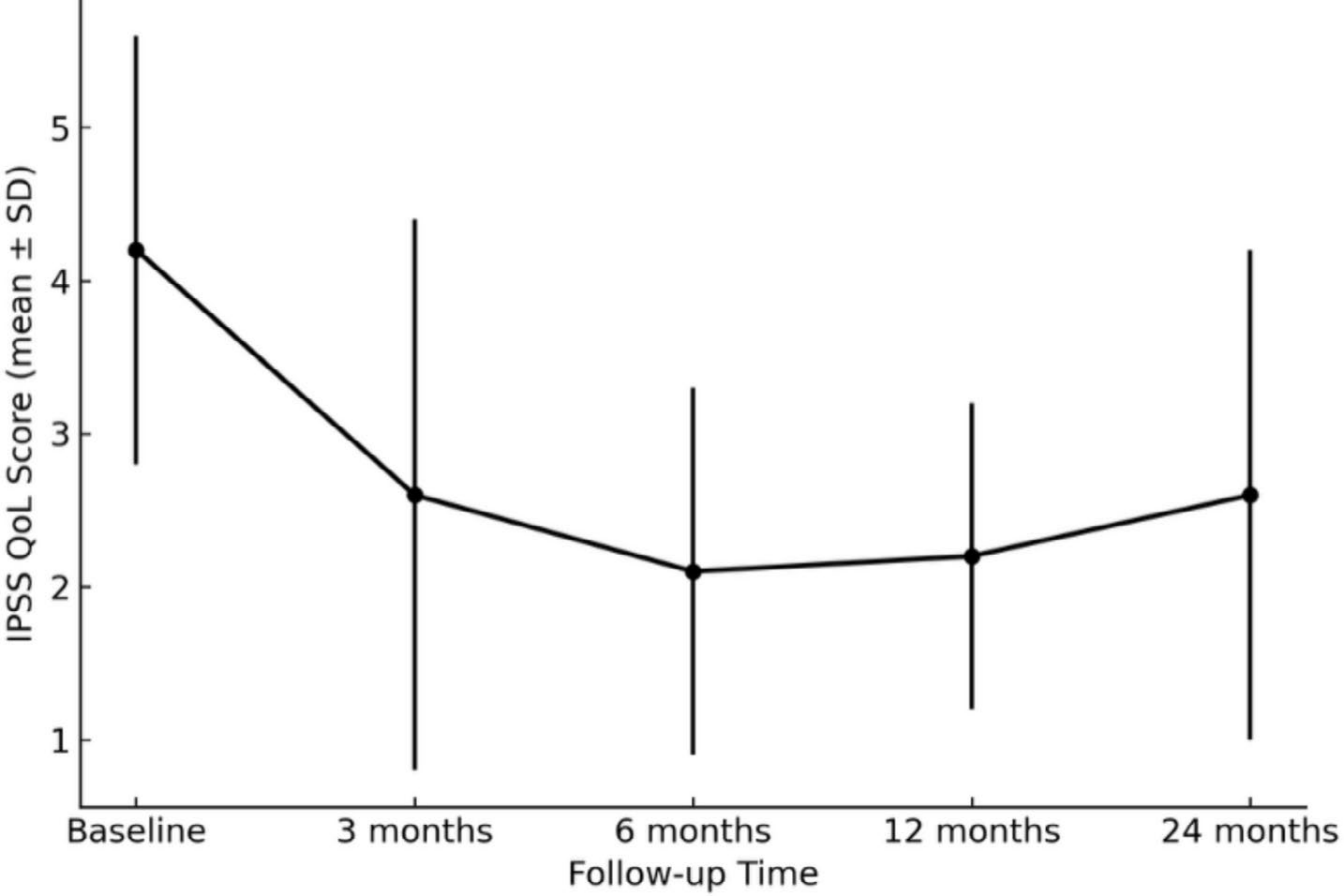
IPSS QoL**—**International prostate symptom score quality of life. IPSS QoL over time following Rezūm therapy (mean ± SD); *p* < 0.005 versus baseline at 3‐, 6‐, 12‐, and 24‐months.

The International Prostate Symptom Score (IPSS) improved significantly, decreasing from 19.8 ± 7.4 at baseline to 11.6 ± 6.3 at 3 months, 9.7 ± 4.9 at 6 months, 10.2 ± 4.6 at 12 months, and 12.4 ± 7.9 at 24 months (Figure 1). The IPSS quality‐of‐life (QoL) score also improved, declining from 4.2 ± 1.8 at baseline to 2.6 ± 1.8, 2.1 ± 1.2, 2.2 ± 1.0, and 2.6 ± 1.5 at the respective follow‐up intervals (Figure 2). Similarly, the Benign Prostatic Hyperplasia Impact Index (BPHII) decreased from 7.2 ± 3.4 at baseline to 3.9 ± 3.3 at 3 months, 3.4 ± 3.7 at 6 months, 3.5 ± 2.1 at 12 months, and 4.0 ± 4.5 at 24 months.

The mean peak urinary flow rate (Qmax) increased from 8.5 ± 5.8 mL/s at baseline to 14.4 ± 1.7 mL/s at 3 months, 16.8 ± 2.5 mL/s at 6 months, 14.0 ± 3.5 mL/s at 12 months, and 15.0 ± 4.0 mL/s at 24 months. Post‐void residual volume (PVR) decreased from 115.5 ± 93.7 mL at baseline to 73.6 ± 54.9 mL at 3 months, 67.8 ± 39.0 mL at 6 months, 54.6 ± 39.7 mL at 12 months, and 42.8 ± 29.2 mL at 24 months (Table 2). However, neither Qmax nor PVR demonstrated a statistically significant change at any time point.

**TABLE 2:** Functional outcomes at baseline and follow‐up.

Variable	Measure	Baseline	M03	M06	M12	M24
IPSS	*n*	44	31	23	23	16
	Baseline	19.8 ± 7.4	21.1 ± 7.3	21.4 ± 6.1	21.6 ± 5.5	21.2 ± 5.8
	Follow Up		11.6 ± 6.3	9.7 ± 4.9	10.2 ± 4.6	12.4 ± 7.9
	Change		−9.4 ± 8.4	−11.7 ± 6.5	−11.4 ± 7.1	−8.9 ± 9.5
	% Change		−44.7	−54.9	−52.9	−41.8
	*p* value		< 0.001	< 0.001	< 0.001	0.007
IPSS QoL	*n*	44	31	23	23	16
	Baseline	4.2 ± 1.4	4.4 ± 1.3	4.4 ± 1.3	4.4 ± 1.2	4.4 ± 1.2
	Follow Up		2.6 ± 1.8	2.1 ± 1.2	2.2 ± 1.0	2.6 ± 1.6
	Change		−1.7 ± 2.1	−2.3 ± 1.6	−2.3 ± 1.4	−1.8 ± 1.6
	% Change		−40.0	−52.5	−51.0	−40.8
	*p* value		< 0.001	< 0.001	< 0.001	0.002
BPHII	*n*	36	20	11	14	6
	Baseline	7.2 ± 3.4	6.8 ± 3.7	7.8 ± 3.7	7.1 ± 3.7	8.5 ± 2.5
	Follow Up		3.9 ± 3.3	3.4 ± 3.7	3.5 ± 2.1	4.0 ± 4.5
	Change		−2.9 ± 4.4	−4.5 ± 4.3	−3.6 ± 3.3	−4.5 ± 5.0
	% Change		−42.6	−57.0	−51.0	−52.9
	*p* value		0.013	0.013	0.003	0.104
Qmax (mL/s)	*n*	40	5	3	4	3
	Baseline	8.5 ± 5.8	10.1 ± 5.3	7.9 ± 2.4	7.4 ± 2.2	7.9 ± 2.4
	Follow Up		14.4 ± 1.7	16.8 ± 2.5	14.0 ± 3.5	15.0 ± 0.0
	Change		4.3 ± 6.9	8.9 ± 3.4	6.6 ± 3.7	7.1 ± 2.4
	% Change		43.1	112.2	87.9	89.1
	*p* value		0.312	0.250	0.125	0.250
PVR (mL)	*n*	44	8	6	17	6
	Baseline	115.5 ± 93.7	162.8 ± 87.7	139.5 ± 92.6	117.5 ± 100.0	129.7 ± 103.5
	Follow Up		73.6 ± 54.9	67.8 ± 39.0	54.6 ± 39.7	42.8 ± 29.2
	Change		−89.1 ± 83.0	−71.7 ± 95.1	−62.9 ± 89.9	−86.8 ± 97.5
	% Change		−54.8	−51.4	−53.6	−67.0
	*p* value		0.022	0.156	0.020	0.141

*Note:* Changes in outcomes from baseline to 3‐, 6‐, 12‐, and 24‐months follow‐up.

Abbreviations: BPHII, Benign Prostatic Hyperplasia Impact Index; IPSS, International Prostate Symptom Score; PVR, post‐void residual volume; Qmax, peak urinary flow; QoL, quality of life; SD, standard deviation.

### 3.2 Adverse Event

Adverse events were observed in a subset of patients. Urinary tract infection occurred in 5 patients (8.6%), epididymitis in 3 patients (5.2%), and acute urinary retention in 2 patients (3.4%), with only one requiring hospitalization. No thrombotic events were identified, although 19 patients (32.8%) were receiving antithrombotic therapy during the perioperative period. No patient developed catheter dependency after treatment, and no further adverse events were documented during the study period.

## 4 Discussion

The number of studies focusing on the very old population (aged > 80 years) has increased significantly since 2000, with a notable surge beginning in the mid‐2000s [13]. However, there remains a notable lack of publications specifically addressing the use of Rezūm therapy in patients aged over 80 years.

A systematic review evaluating minimally invasive surgical therapies for benign prostatic hyperplasia in the geriatric population screened 292 studies. Among the procedures analyzed, Rezūm demonstrated one of the most favorable safety profiles, with the widest reported safety margin compared to HoLEP, ThuLEP, and DiLEP [14]. In our study, Rezūm was also associated with a low rate of postoperative adverse events, with only a single hospitalization reported.

Also, a recent Italian study comparing men younger than 75 with those older than 75 found no significant differences between the groups in terms of hospital length of stay, postoperative urinary retention, or reintervention rates [15]. Similarly, a single‐center study of patients treated with Rezūm therapy, stratified into younger (< 65 years) and older (≥ 65 years) cohorts, reported no significant differences in International Prostate Symptom Score (IPSS), quality of life (QoL), or adverse events (AEs) over a four‐year follow‐up period [16].

When focusing specifically on patients over 80 years of age, a study included 34 individuals with a mean age of 85.4 ± 5.1 years and reported statistically significant clinical improvements at 6 months post‐procedure. The mean IPSS score improved from 21.7 ± 4.0 at baseline to 12.6 ± 2.2; the IPSS‐related quality of life (QoL) score decreased from 4.4 ± 0.9 to 2.2 ± 0.7; the peak urinary flow rate (Qmax) increased from 7.0 ± 3.3 mL/s to 15.0 ± 1.2 mL/s; and the post‐void residual volume (PVR) decreased from a median of 125.0 mL (IQR: 82.5–226.0) to 45.2 mL (IQR: 17.5–63.5) [17].

In our cohort, we observed that these clinical improvements were sustained over a longer follow‐up period. The IPSS significantly improved from a baseline of 19.8 ± 7.4 to 11.6 ± 6.3 and remained stable at 12.4 ± 7.9 at 24 months. Similarly, the QoL score decreased from 4.2 ± 1.8 at baseline to 2.6 ± 1.5 at 24 months. The mean Qmax increased from 8.5 ± 5.8 mL/s to 15.0 ± 4.0 mL/s, while the mean PVR declined from 115.5 ± 93.7 mL to 42.8 ± 29.2 mL over the same period.

Our study demonstrated that Rezūm therapy is a viable option for very elderly patients, providing significant improvements in their quality of life. These findings are supported by previous research confirming that Rezūm is a safe, effective, and minimally invasive treatment for LUTS/BPH in individuals aged 80 years and older, including those with urinary retention or substantial comorbidities who may not be suitable candidates for conventional surgical interventions [18]. Indubitably, patients over 80 years of age warrant special consideration due to their overall health status and anesthetic risk. In this cohort, Rezūm therapy demonstrated excellent feasibility, as most procedures (72.4%) were successfully performed under propofol‐based intravenous sedation in an outpatient setting. This underscores a major advantage of Rezūm—its ability to be safely performed in frail elderly patients with minimal anesthesia requirements and rapid postoperative recovery.

The main limitation of this study is the relatively small sample size and its restriction to two high‐volume centers, which may limit the generalizability of the findings to broader clinical settings or more diverse patient populations. While follow‐up extended to 24 months, not all patients completed long‐term assessments, introducing the potential for attrition bias. This was particularly evident for Qmax and PVR measurements, which required patients to return to the clinic for in‐person testing. Given that participants were recruited from different geographic regions in Italy and Canada, logistical challenges and travel distance contributed to incomplete follow‐up for these parameters. Additionally, while key functional outcomes such as IPSS, QoL, Qmax, and PVR were thoroughly evaluated, more detailed data on sexual function, patient satisfaction, and frailty status were not systematically collected. In particular, data regarding sexual function and ejaculation were inconsistently reported during follow‐up, representing a further limitation.

Although detailed comorbidity or frailty indices were not systematically available across centers, the presence of multiple chronic conditions such as hypertension, diabetes, and the frequent use of antithrombotic therapy underscore the high comorbidity burden in this cohort. Despite this, perioperative outcomes were favorable, with no thrombotic or major cardiovascular events observed. These findings support the safety of Rezūm therapy even among very elderly and medically complex patients, a population often considered suboptimal candidates for more invasive surgical interventions.

## 5 Conclusion

Rezūm water vapor thermal therapy is a safe, effective, and minimally invasive option for treating lower urinary tract symptoms (LUTS) secondary to benign prostatic hyperplasia (BPH) in patients aged 80 years and older. The procedure was well tolerated and associated with a low incidence of adverse events.

## Author Contributions

Study concept and design: Mario Bitar, Sagi Shprits. Data acquisition: Dean Elterman. Data analysis: Katherine Lajkosz. Drafting of manuscript: Mario Bitar. Critical revision of the manuscript: Naeem Bhojani, Bilal Chughtai, Kevin C. Zorn, Luca Cindolo, Giovanni Ferrari.

## Funding

The authors have nothing to report.

## Conflicts of Interest

Dr. Dean Elterman is a consultant/investigator for Boston Scientific, Procept Biorobotics, Olympus, Urotronic, Prodeon, and Zenflow. Dr. Bilal Chughtai is a consultant for Boston Scientific, Olympus, and an investigator for Teleflex and Abbvie. Dr. Naeem Bhojani is a consultant/investigator for Boston Scientific, Procept BioRobotics, and Olympus. The remaining authors declare no potential conflicts of interest.

## Data Availability

The data that support the findings of this study are available on request from the corresponding author. The data are not publicly available due to privacy or ethical restrictions.
